# Oncological and reproductive outcomes of conization combined with pelvic node evaluation in patients with early-stage cervical cancer: a systematic review and meta-analysis

**DOI:** 10.3389/fonc.2023.1251453

**Published:** 2023-09-19

**Authors:** Yisi Wang, Yali Chen, Mengyao Wang, Zhaojuan Qin, Lingli Zhang, Ai Zheng, Ling Han

**Affiliations:** ^1^ Department of Obstetrics and Gynecology, West China Second University Hospital, Sichuan University, Chengdu, Sichuan, China; ^2^ Key Laboratory of Birth Defects and Related Diseases of Women and Children (Sichuan University), Ministry of Education, Chengdu, Sichuan, China

**Keywords:** conization, pelvic node evaluation, oncological outcome, reproductive outcome, cervical cancer

## Abstract

**Objective:**

This study aims to preliminarily assess the oncological and reproductive outcomes of fertility preservation treatment using conization combined with pelvic node evaluation in young patients with early-stage cervical cancer (ECC) through meta-analysis.

**Methods:**

In this meta-analysis, we analyzed studies published in PubMed, Embase, Cochrane Central Register of Controlled Trials (CENTRAL), International Clinical Trials Registry Platform (ICTRP), and Clinical Trials. gov that appeared in our search from inception to 0 7/02/2023.

**Results:**

There were 17 relevant studies with a total of 620 patients included, of which 444 patients received conization combined with pelvic node evaluation. The combined pregnancy rate was 45.4% (95% CI: 0.34–0.57), the combined live birth rate was 33.9% (95% CI: 0.26–0.42), the combined miscarriage rate was 4.8% (95% CI: 0.02–0.092), the combined preterm delivery rate was 5.1% (95% CI: 0.02–0.092), and the combined recurrence rate was 1.9% (95% CI: 0.006–0.035), which did not significantly differ from that of patients who received radical surgery (OR: 0.689, 95% CI: 0.506–0.938).

**Conclusion:**

Cervical conization combined with pelvic lymph node evaluation for fertility preservation in young ECC patients can achieve oncological outcomes similar to radical surgery while improving pregnancy success rates and preserving postoperative fertility. In summary, fertility preservation treatment using cervical conization combined with pelvic lymph node evaluation may be considered as a viable option for young ECC patients with strong fertility preservation desire, resulting in better pregnancy and live birth outcomes.

**Systematic review registration:**

https://www.crd.york.ac.uk/PROSPERO/#myprospero, identifier PROSPERO (CRD42023423432).

## 1 Introduction

Cervical cancer is the fourth most common female malignancy worldwide (1). With the widespread use of HPV and cervical cancer cell screening, the detection rate of early cervical cancer has greatly increased. At the same time, the morbidity of young patients is gradually increasing due to changes in social lifestyle. It has been reported that around 35% of cervical cancer patients are under 40 years old (2) and a considerable proportion of them have not completed childbirth or still have fertility requirements. Currently, first-line treatment advised by guidelines for ECC is radical hysterectomy with bilateral pelvic lymphadenectomy and/or sentinel lymph node (SLN) biopsy with or without salpingo-oophorectomy (3), which results in loss of fertility and is not acceptable for young patients.

In ECC, many studies have shown that the incidence of parametrial involvement (PI) is low in patients with tumor size < 2 cm, negative pelvic lymph nodes, and invasion depth < 10 mm (4, 5). This supports the use of simpler fertility-preserving surgical methods for young patients with small tumor volume and limited local lesions, further improving their quality of life. For women with ECC who want to preserve fertility, both FIGO and NCCN guidelines recommend conization with lymph node evaluation for stage IA1 no lymphovascular space invasion (LVSI), radical trachelectomy or conization with lymph node evaluation for stage IA1 with LVSI and stage IA2, or radical trachelectomy with lymph node evaluation for stage IB1 and selected IB2 (6, 7).

Dargent et al. published his experience of performing RT with laparoscopic pelvic lymph node dissection for young women with ECC in 1994 (8, 9). Some studies show that RT is a safe and feasible technique with similar oncological results to cervical conization, but it has a high rate of miscarriage and preterm labor during pregnancy (10, 11), which may impair postoperative reproductive outcomes. Several studies have reported that conization has generally favorable obstetric outcomes compared with RT (12, 13). Although current guidelines recommend the application of cervical conization in ECC, the safety, feasibility, and treatment outcome of conization combined with lymph node evaluation in patients with ECC have not been fully evaluated.

We conducted a systematic review to evaluate the oncological and fertility outcomes of using cervix conization combined with pelvic lymph node evaluation surgery to treat ECC patients.

## 2 Materials and methods

The systematic review and meta-analysis were conducted according to the Preferred Reporting Items for Systematic Reviews and Meta-Analysis (PRISMA) guidelines and registered in PROSPERO (CRD42023423432).

### 2.1 Search strategy

We systematically examined the PubMed, EMBASE, Cochrane Library, International Clinical Trials Registry Platform (ICTRP), and Clinical Trials electronic databases to 07/02/2023, to identify relevant literature reporting the use of cervix conization combined with pelvic lymph node evaluation surgery for fertility preservation in patients with ECC. These studies reported the oncological and fertility outcomes of ECC patients. The following search terms were used to identify relevant studies on early cervical cancer: “cervical cancer” and “cervical carcinoma,” whereas the following terms were used to identify relevant studies on conization: “cone biopsy” and “conization”. The following terms were used to identify relevant studies on pelvic lymph node evaluation: “lymph node assessment,” “lymph node dissection,” “lymph node evaluation,” “lymph node excision,” “lymphadenectomy*,” and “lymphadenectomy”. The search was limited to English-language publications. We rigorously reviewed the reference lists of all the articles identified in our search based on inclusion and exclusion criteria, to identify any potentially missing studies or unpublished data. If multiple studies analyzed overlapping patient populations, we selected the most recent or comprehensive results.

### 2.2 Inclusion and exclusion criteria

The inclusion criteria included the following: (1) Primary cervical cancer patients who received conization combined with lymph node evaluation as initial treatment options were included. (2) The average age of patients included in the literature was less than 40 years old. (3) The clinical stage was FIGO IA1-IB1 (2018 FIGO staging). (4) Tumor diameter <2 cm. (5) No other tumors combined or history of other tumor treatments.

Exclusion criteria included the following: (1) Pathological types were cervical neuroendocrine tumors. (2) Postoperative pathology combined with endometrial cancer or other tumors. (3) Malignant tumors of other tissue sites or metastatic cervical tumor. (4) Literature that did not analyze and statistically report pregnancy and oncological outcomes, without a clearly defined follow-up deadline or an unreasonable experimental design. (5) Fertility-damaging treatments such as radiotherapy after cone-shaped excision of the cervix combined with pelvic lymph node evaluation surgery for early cervical cancer. (6) Individual case reports or literature with repetitive data (the literature with the latest or more comprehensive results was used for repetitive data). (7) Literature with fewer than five cases.

### 2.3 Study selection

Two reviewers (YSW and LLZ) screened the studies initially based on titles and abstracts, removing duplicate studies and those that did not meet the review criteria, and then read the remaining articles in full to include eligible studies. Disagreements were resolved through consultation with a third reviewer (YLC). The quality of included studies was evaluated using the non-randomized studies index (MINORS) (14).

### 2.4 Data extraction and results calculation

Two independent reviewers (MYW and ZJQ) extracted the following data from each study: study author, publication date, study design type (prospective or retrospective), number of patients, median patient age, FIGO stage, tumor histological type, oncological and reproductive outcomes, median follow-up time, and so on.

In this study, we defined pregnancy rate as the number of women who successfully conceived divided by the total number of women who retained fertility during follow-up; live birth rate as the number of surviving infants divided by the total number of women who retained fertility during follow-up; abortion rate as the ratio of women who experienced one or more abortions to the total number of women who retained fertility during follow-up; and premature birth rate as the ratio of women who experienced one or more premature births to the total number of women who retained fertility during follow-up. Recurrence rate was defined as the number of recurrence cases divided by the total number of included patients.

### 2.5 Statistical analysis

The data extracted were statistically summarized and analyzed using Stata 17.0. Random-effects models were calculated using the inverse variance method, and forest plots were generated for each outcome to obtain individual study and pooled estimates with 95% CI (15). *I* ^2^ was used to assess heterogeneity of outcome data (16), and *I* ^2^ >50 was considered high heterogeneity. Sources of heterogeneity were determined through subgroup analysis and sensitivity analysis. Publication bias was assessed using Begg–Mazumdar rank correlation and funnel plots.

## 3 Results

### 3.1 Search results

**Figure 1:**
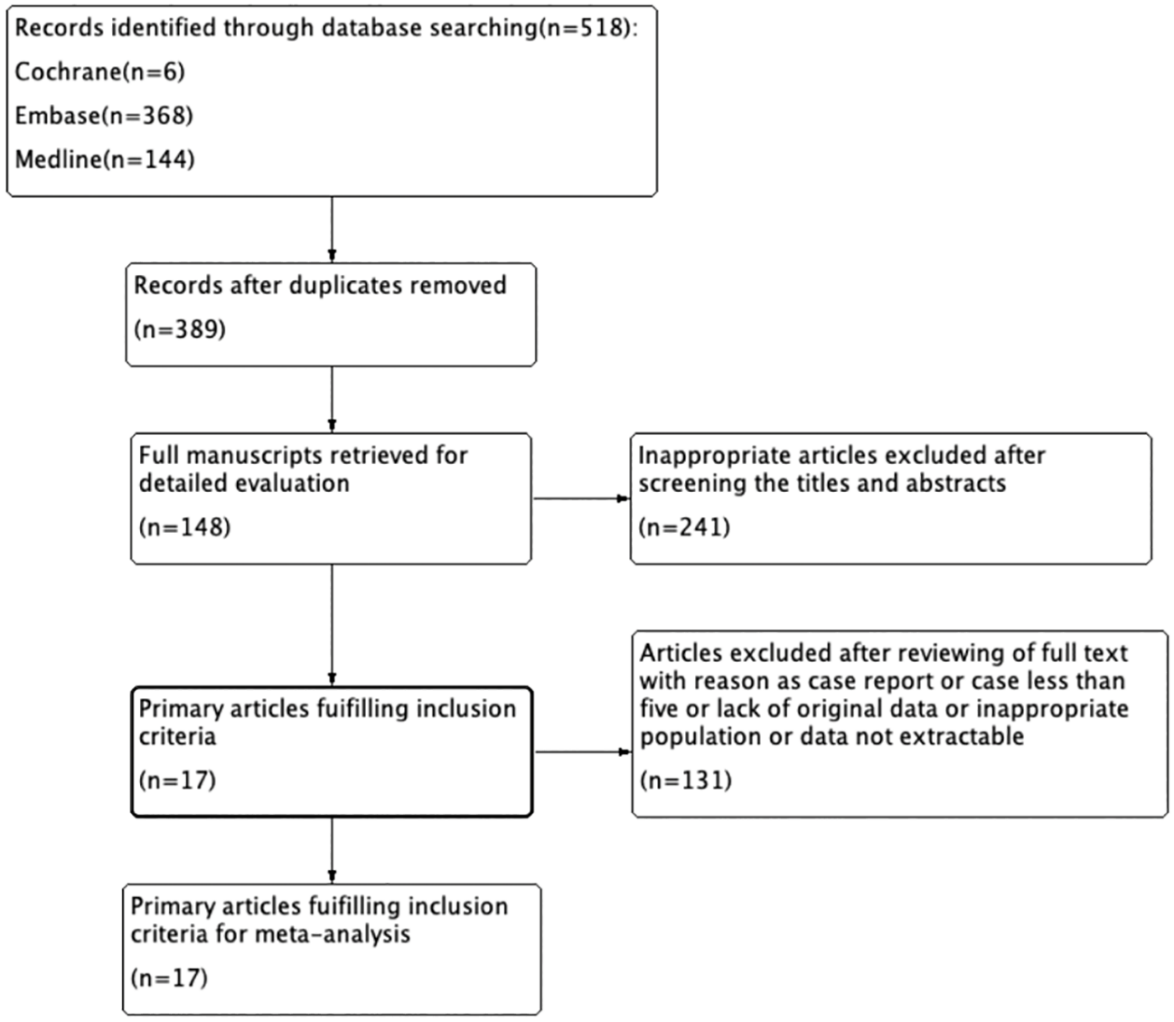
Flowchart of literature selection process.

A total of 518 studies were retrieved through computer databases and manual searches, which were basically in line with the query requirements. After removing 129 duplicate studies, the remaining 389 articles were screened based on titles and abstracts, and obviously ineligible articles were excluded, resulting in a total of 148 articles remaining. After reading the full texts, 17 studies that met the study criteria were eventually included in the analysis (17–33). The specific search process are detailed in **Figure 1**.

### 3.2 Included literature and characteristics of studies

**Figure 2:**
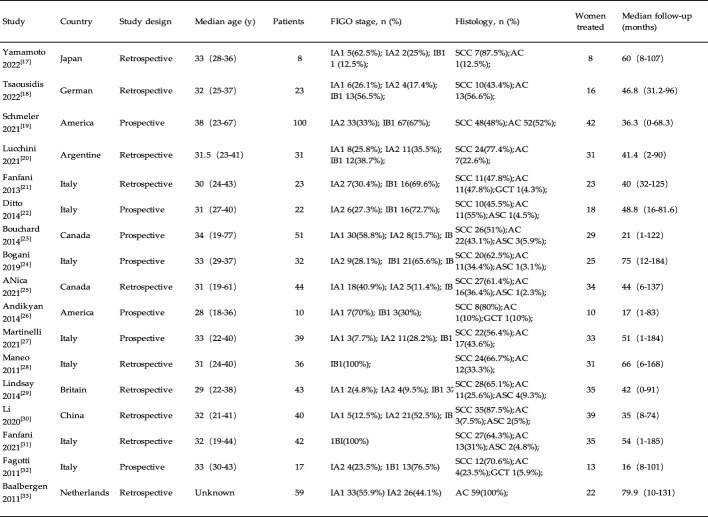
Characteristic of the studies.

A total of 17 English language studies were included in this study, consisting of 7 prospective studies and 10 retrospective studies, including 620 young patients with ECC. These studies were conducted in various countries, including the United States (n = 2), Japan (n = 1), Canada (n = 2), Germany (n = 1), China (n = 1), Italy (n = 7), United Kingdom (n = 1), Argentina (n = 1), and the Netherlands (n = 1). The average age of onset for the included patients in these studies was close (between 29 and 38 years old), and the follow-up time ranging from 16 to 79.9 months. General information of the included literature is shown in **Figure 2**.

### 3.3 Quality assessment of included studies

**Figure 3:**
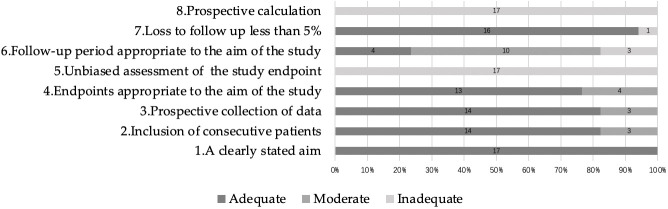
Quality assessment.

This article included a total of 17 English language studies. All included literature was assessed for quality using the non-randomized controlled trials methodological evaluation index: MINORS. All studies had clear objectives, but blinding was not used during the study process and the necessary sample sizes were not prospectively estimated. A total of 14 studies consecutively included patients, 14 studies collected data that was designed in the study protocol before the study began, and 13 studies had endpoints that could adequately reflect the research objectives. According to the guidelines, the follow-up time should be at least 5 years. Only 4 studies out of the 17 studies reported follow-up data for at least 5 years. One study reported a >5 follow-up loss (29). The quality assessment of all studies is shown in **Figure 3**.

### 3.4 Fertility and oncologic outcomes

#### 3.4.1 Pregnancy rates

**Figure 4:**
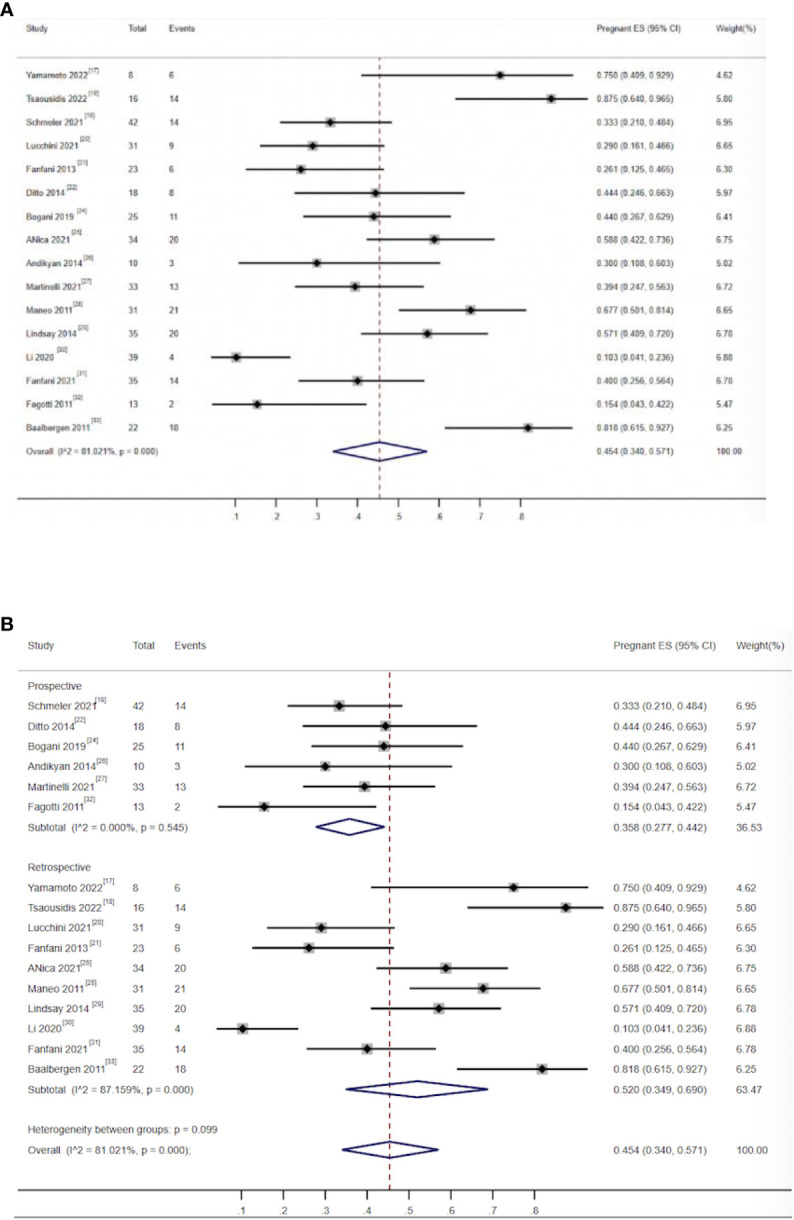
**(A)** Forest plot of the meta-analysis of pregnancy rate in ECC patients who underwent conization combined with pelvic node evaluation. **(B)** Forest plot of the meta-analysis of pregnancy rate in ECC patients who underwent conization combined with pelvic node evaluation using study type subgroup analysis.

There were 16 studies that reported on pregnancy rates, including 569 patients. A total of 415 (72.93%) patients successfully received conization combined with pelvic node evaluation. Furthermore, 183 young women achieved at least one pregnancy, and the combined pregnancy rate was 45.4% (95% CI, 0.34–0.571) (17–22, 24–33). The heterogeneity test result for the included studies was *I* ^2^ = 81.0, P < 0.05 (**Figure 4-1**), indicating high heterogeneity among the included studies. The sensitivity analysis could not identify the source of heterogeneity by eliminating studies one by one. Subgroup analysis showed that when studies were grouped by research type, the combined pregnancy rate for prospective studies was 35.8 (95% CI, 0.227–0.442) (19, 22, 24, 26, 27, 32), I^2^ = 0 P > 0.05 (**Figure 4-2**), indicating that there was no obvious heterogeneity among prospective studies. The source of heterogeneity could not be identified in other subgroups. The type of study directly affects the quality of evidence and therefore the quality of the integration result. The heterogeneity among prospective studies was significantly reduced in subgroup analysis by study type, and the overall sample pregnancy rate should be closer to the data obtained from prospective studies.

#### 3.4.2 Live birth rate and miscarriage rate

**Figure 5:**
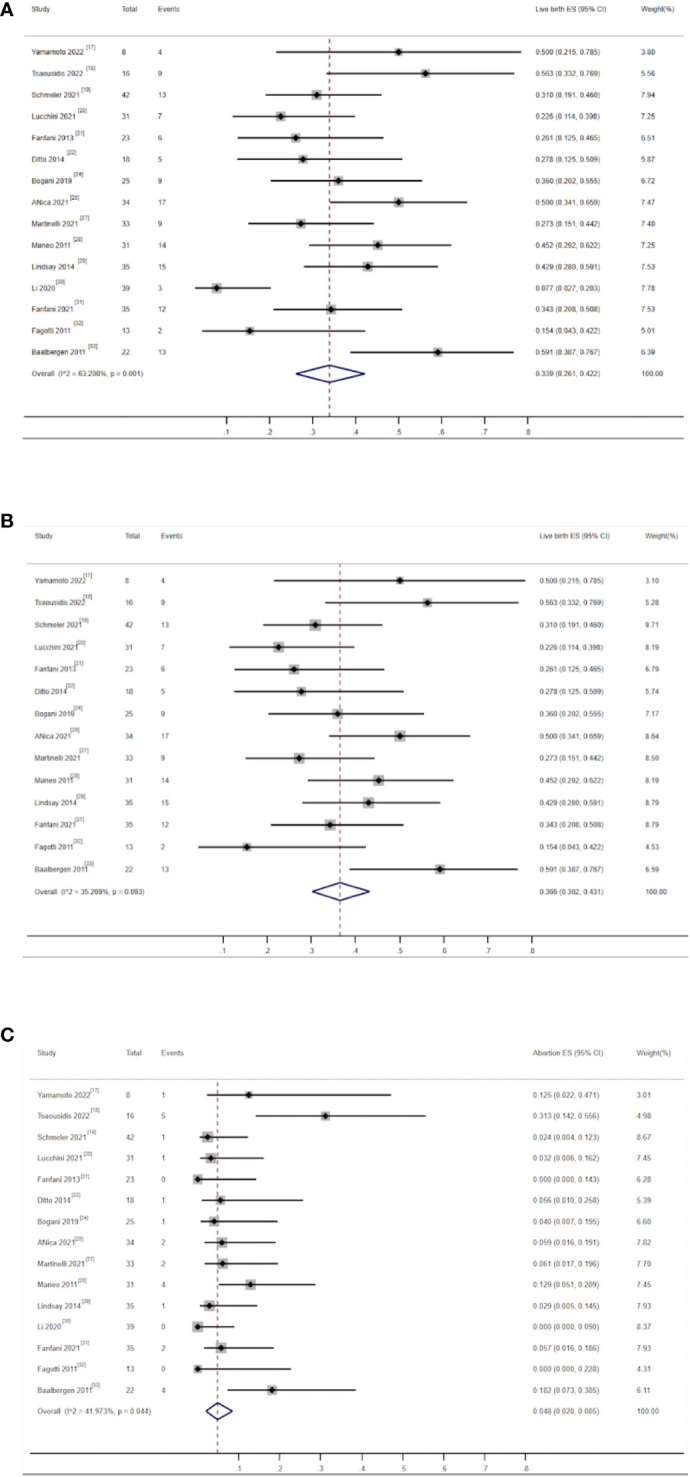
**(A)** Forest plot of the meta-analysis of live birth rate in ECC patients who underwent conization combined with pelvic node evaluation. **(B)** Forest plot of the meta-analysis of live birth in ECC patients who underwent conization combined with pelvic node evaluation, after removal of one study (30). **(C)** Forest plot of the meta-analysis of abortion rate in ECC patients who underwent conization combined with pelvic node evaluation.

There were 15 studies that reported on live birth rates and miscarriage rates, including 559 patients. Among them, 405 (72.45%) patients successfully received fertility-preserving treatment. Among them, 138 women gave birth to at least one healthy baby. The combined live birth rate was 33.9% (95% CI, 0.261–0.422) (17–22, 24–33), and the heterogeneity test result for the included studies was I^2^ = 63.2 P < 0.05, indicating high heterogeneity among the included studies (**Figure 5-1**). Subgroup analysis showed no significant difference among subgroups. Sensitivity analysis identified one study as a potential source of heterogeneity (30). Excluding it greatly reduced heterogeneity (I^2^ = 35.26 P > 0.05) and yielded a similar outcome as the combined live birth rate in all studies [36.6% (0.302–0.431)] (**Figure 5-2**). There were 25 patients who had experienced miscarriage once or more. The combined miscarriage rate was 4.8% (95% CI, 0.02–0.085), and the heterogeneity test result for the included studies was I^2^ = 41.97 P = 0.044, indicating low heterogeneity among the included studies (**Figure 5-3**).

#### 3.4.3 Preterm delivery rate

**Figure 6:**
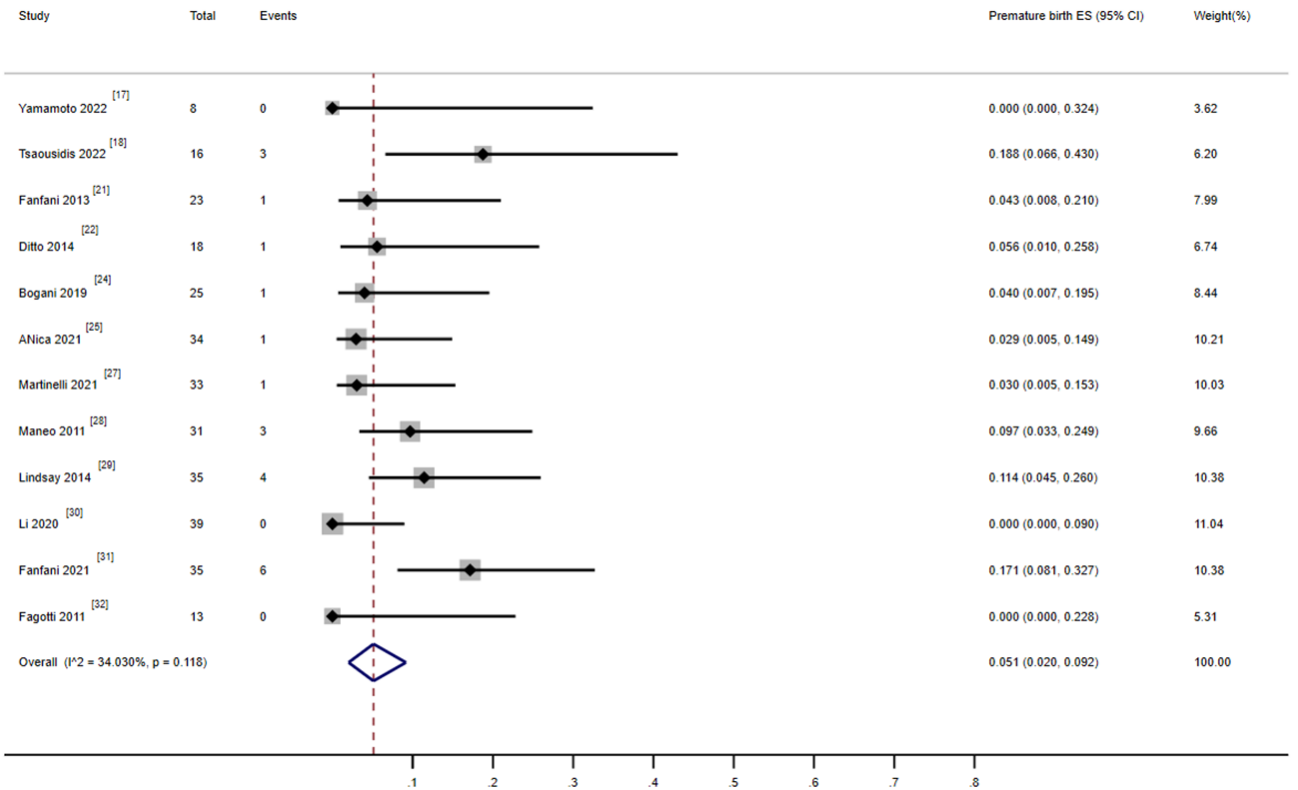
Forest plot of the meta-analysis of premature rate in ECC patients who underwent conization combined with pelvic node evaluation.

There were 12 studies that reported on the preterm delivery rate, including 369 patients. Among them, 310 (84%) patients successfully received fertility-preserving treatment (17, 18, 21, 22, 24, 25, 27–32). Among them, 21 women experienced at least one preterm delivery. The combined preterm delivery rate was 5.1% (95%, 0.02–0.092), and the heterogeneity test result for the included studies was I^2^ = 34.03 P > 0.05, indicating low heterogeneity among the included studies (**Figure 6**).

#### 3.4.4 Recurrence rate

**Figure 7:**
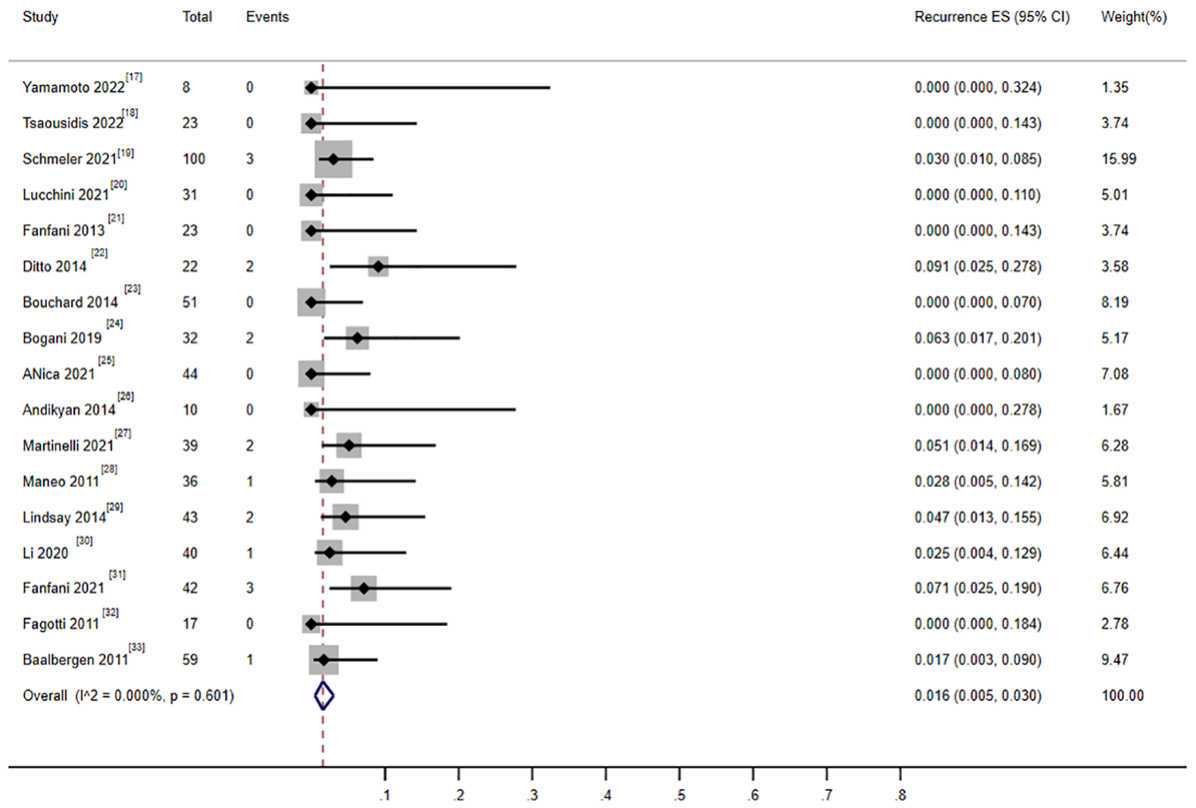
Forest plot of the meta-analysis of recurrence rate in ECC patients who underwent conization combined with pelvic node evaluation.

There were 17 studies that reported on the recurrence rate, including 620 patients. Moreover, 18 patients experienced recurrence, and the combined recurrence rate was 1.9% (0.006–0.035) (17–33) (**Figure 7**). The heterogeneity test result for the included studies was I^2^ = 0 P > 0.05, indicating no significant heterogeneity among the included studies. Three studies involving 210 patients reported recurrence rates of patients who received cervical conization combined with pelvic lymph node evaluation (1.05% 1/95) or radical surgery (2.6% 3/115) (18, 23, 33). The ratio between the two groups showed no significant difference (OR = 0.689, 0.506–0.938).

#### 3.4.5 Publication bias

**Figure 8:**
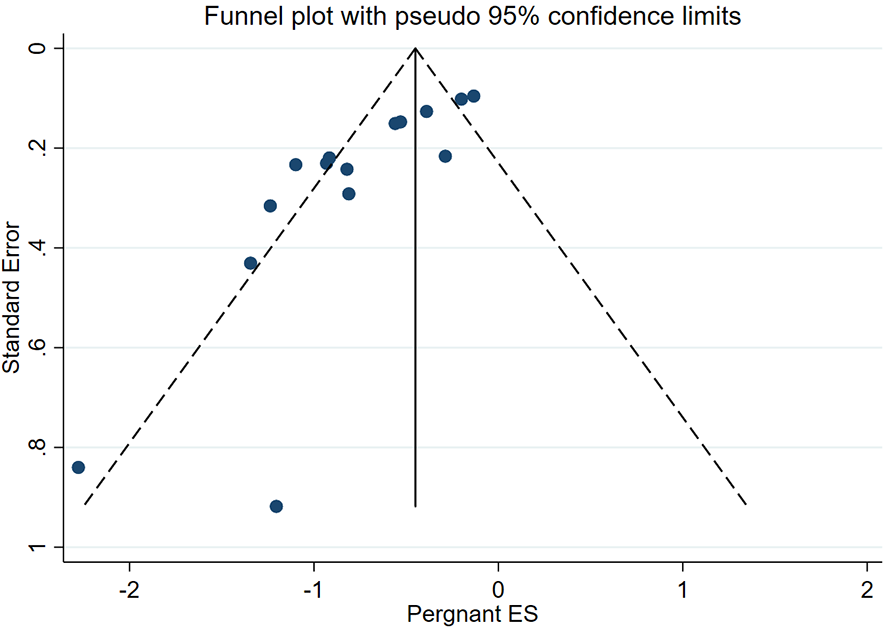
Funnel plot of the meta-analysis of pregnancy rate in ECC patients who underwent conization combined with pelvic node evaluation.

Begg–Mazumdar rank correlation test showed that the funnel plot in the meta-analysis of the main outcome indicator pregnancy rate in ECC patients undergoing cervical conization combined with pelvic lymph node evaluation is slightly asymmetric (**Figure 8**), indicating the possibility of publication bias in the corresponding study. This phenomenon may be due to the inadequate retrieval of negative results in literature or biased database literature inclusion criteria, which to some extent weakened the reliability of the statistical results.

## 4 Conclusions

Fertility preservation is becoming an increasingly important issue for young cervical cancer patients. While ensuring the outcome of oncology, the reproductive outcome should be further improved. The results of this study clarify that cervical conization combined with pelvic lymph node evaluation can achieve similar oncological outcomes as RT for ECC while also achieving more optimal obstetric outcomes.

Cervical conization combined with pelvic lymph node evaluation showed good results in terms of oncological outcomes. Nezhat et al.’s study has shown that among all fertility-sparing treatments with or without pelvic node evaluation, the overall mean cancer recurrence rate was 3.2% (34). Rob et al.’s study has shown that the recurrence rate of Dargent RT surgery is between 4.2% and 4.7% (35), and Plante summarized the recurrence rates of abdominal and laparoscopic RT as 4% and 7%, respectively (36). In our meta-analysis, the recurrence rates mentioned in the 17 articles were very low, ranging from 0% to 9.1% with a combined recurrence rate of 1.6% (95% CI, 0.005–0.03) indicating comparable recurrence results with RT. Some studies have shown that 60% of patients who underwent RT did not have residual tumor lesions in the surgical specimens, indicating that these patients can be treated with less aggressive surgery to achieve the expected oncological outcomes (37). Based on these results, we recommend that cervical conization combined with pelvic lymph node evaluation be considered as a safe alternative to RT for young women with ECC who wish to preserve fertility.

In terms of reproductive outcomes, RT is often reported to increase the risk of postoperative premature birth and miscarriage, reducing the success rate of postoperative fertility preservation in young patients. Pareja et al. summarized the pregnancy rates after RT for ECC via abdominal and vaginal routes globally to be 16.2% and 24% (38). Additionally, some studies have found high rates of miscarriage in early and middle pregnancy after RT (16%–20% and 8%–10%), with a high risk of premature birth (20%–30%) (10, 11, 39). In our meta-analysis, the combined results of cervical conization combined with pelvic lymph node evaluation seem to be more ideal for reproductive outcomes. In ECC patients undergoing cervical conization combined with pelvic node evaluation, approximately half (45.4%) of the patients can conceive, with as high as one-third (33.9%) giving birth to healthy babies and only 4.8% experiencing miscarriage and 5.1% experiencing premature birth. This may be mainly due to the relatively minor removal of para-cervical tissue and less damage to pelvic floor function during the surgery.

However, our meta-analysis has the following limitations: Significant heterogeneity was observed among studies in the analysis of pregnancy rate and live birth rate, reflecting the differences between included studies. The retrospective design and differences in sample size of the studies may also be sources of heterogeneity. We were able to identify individual studies with significant contributions to heterogeneity, and exclusion of these studies for repeat analysis yielded similar results to the original analysis. Our study was conducted through ratio-based rather than randomized controlled trials, which may introduce many confounding effects and weaken the reliability of evidence. The inclusion of only English-language studies may also introduce biases, and the limited availability of domestic research data in this study may differ from China’s genetics, environment, and health conditions. Therefore, whether this treatment method is beneficial to domestic cervical cancer patients in preserving fertility while ensuring survival rate or specific indications still requires further verification.

Although the above limitations exist, our meta-analysis results indicate that cervical combined with pelvic lymph node evaluation has a good oncological and reproductive outcome. To our knowledge, this is the first meta-analysis to evaluate the oncological and reproductive outcomes after cervical conization combined with pelvic lymph node evaluation.

Cervical conization with pelvic lymph node evaluation seems to be an acceptable treatment for well-selected patients with low-risk, early-stage cervical cancer who wish to preserve fertility. It offers excellent oncological outcomes and good reproductive results. Further large prospective studies are warranted to prove the effectiveness of this surgery.

## Data availability statement

The original contributions presented in the study are included in the article/supplementary material. Further inquiries can be directed to the corresponding authors.

## Author contributions

YW: conceptualization, data curation; YC: writing—original draft preparation; LZ: methodology, software, validation; MW: visualization, investigation; ZQ: methodology, formal analysis; LH: supervision: AZ: writing—review editing. All authors contributed to the article and approved the submitted version.
